# Diphenyl chloro­thio­phospho­nate

**DOI:** 10.1107/S160053681104030X

**Published:** 2011-10-08

**Authors:** Yan-Fei Zhang, Pei-Hua Zhao, Jun-Jie Liu, Gui-Zhe Zhao

**Affiliations:** a Research Center for Engineering Technology of Polymeric Composites of Shanxi Province, School of Materials Science and Engineering, North University of China, Taiyuan 030051, People’s Republic of China

## Abstract

The complete mol­ecule of the title compound, C_12_H_10_ClO_2_PS, is generated by crystallographic mirror symmetry, with the P, S and Cl atoms lying on the mirror plane. The resulting PO_2_SCl tetra­hedron is significantly distorted [O—P—O = 96.79 (9)°]. The crystal packing exhibits no directional inter­actions.

## Related literature

For the application of related compounds as pesticides, see: Greenhalgh *et al.* (1980 ▶); Um *et al.* (2003 ▶).
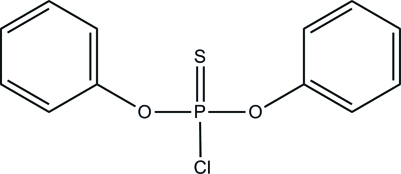


## Experimental

### 

#### Crystal data

C_12_H_10_ClO_2_PS
*M* *_r_* = 284.68
Orthorhombic, 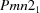

*a* = 14.9779 (18) Å
*b* = 7.3709 (10) Å
*c* = 5.8157 (10) Å
*V* = 642.06 (16) Å^3^
*Z* = 2
Mo *K*α radiation
μ = 0.57 mm^−1^
*T* = 113 K
0.26 × 0.20 × 0.16 mm

#### Data collection

Rigaku Saturn724 CCD diffractometer
Absorption correction: multi-scan (*CrystalClear*; Rigaku/MSC, 2005 ▶) *T*_min_ = 0.866, *T*_max_ = 0.914
6462 measured reflections
1590 independent reflections
1422 reflections with *I* > 2σ(*I*)
*R*_int_ = 0.046

#### Refinement

*R*[*F* ^2^ > 2σ(*F* ^2^)] = 0.026
*wR*(*F* ^2^) = 0.059
*S* = 1.01
1590 reflections
83 parameters
1 restraint
H-atom parameters constrained
Δρ_max_ = 0.33 e Å^−3^
Δρ_min_ = −0.28 e Å^−3^
Absolute structure: Flack (1983 ▶), 716 Friedel pairs
Flack parameter: −0.25 (7)

### 

Data collection: *CrystalClear* (Rigaku/MSC, 2005 ▶); cell refinement: *CrystalClear*; data reduction: *CrystalClear*; program(s) used to solve structure: *SHELXS97* (Sheldrick, 2008 ▶); program(s) used to refine structure: *SHELXL97* (Sheldrick, 2008 ▶); molecular graphics: *SHELXTL* (Sheldrick, 2008 ▶); software used to prepare material for publication: *CrystalStructure* (Rigaku/MSC, 2005 ▶).

## Supplementary Material

Crystal structure: contains datablock(s) global, I. DOI: 10.1107/S160053681104030X/hb6430sup1.cif

Structure factors: contains datablock(s) I. DOI: 10.1107/S160053681104030X/hb6430Isup2.hkl

Supplementary material file. DOI: 10.1107/S160053681104030X/hb6430Isup3.cml

Additional supplementary materials:  crystallographic information; 3D view; checkCIF report

## Figures and Tables

**Fig. 1.**
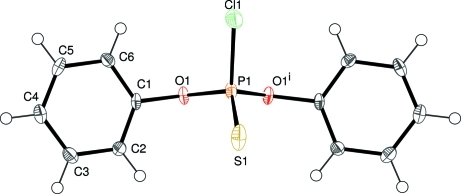
The molecular structure of (I) showing 50% displacement ellipsoids. Symmetry code: (i) –x, y, z.

**Fig. 2.**
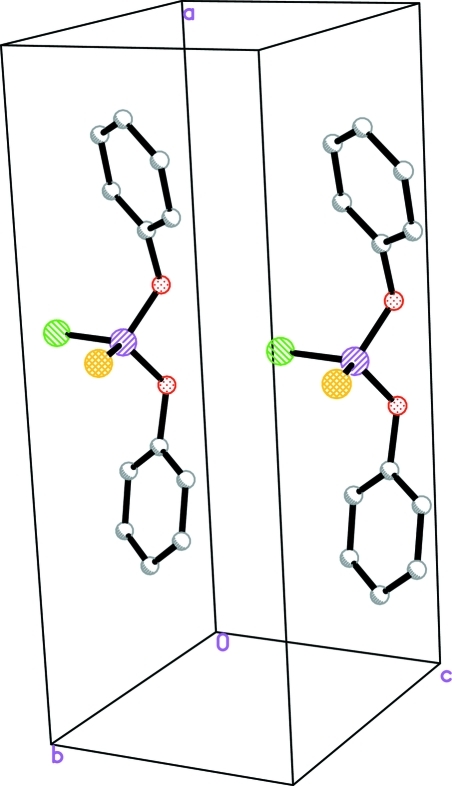
The crystal packing for (I).

## supplementary crystallographic information

### Experimental

Triethylamine (127.0 mmol) were added to the dichloromethane solution (80.0 ml) of phenol (120.0 mmol) while stirring. Thiophosphory chloride (60.0 mmol) was slowly dropwise added to the above solution, and then the rection mixture was refluxed. After the reaction was completed, it is cooled to room temperature. The reaction mixture was washed with water and brine, respectively. The separated organic phase was dried with anhydrous sodium sulfate, and then the solvents were evporated thoroughly *in vacuo*. The obtained crude was separated through column chromatography on silica gel to give the white product. Colourless prisms of the title compound were obtained by slow evaporation of the dichloromethane/n-hexane solutions at room temperature. ^31^P NMR(161.9 MHz, CDCl_3_, TMS): 58.73 (*s*) p.p.m..

### Refinement

All the H atoms were positioned geometrically (C—H = 0.95 Å) and refined as riding with *U*_iso_(H) = 1.2*U*_eq_(C).

### Crystal data

**Table:**

C_12_H_10_ClO_2_PS	*D*_x_ = 1.473 Mg m^−^^3^
*M**_r_* = 284.68	Mo *K*α radiation, λ = 0.71073 Å
Orthorhombic, *P**m**n*2_1_	Cell parameters from 2338 reflections
*a* = 14.9779 (18) Å	θ = 2.7–28.0°
*b* = 7.3709 (10) Å	µ = 0.57 mm^−^^1^
*c* = 5.8157 (10) Å	*T* = 113 K
*V* = 642.06 (16) Å^3^	Prism, colorless
*Z* = 2	0.26 × 0.20 × 0.16 mm
*F*(000) = 292	

### Data collection

**Table:**

Rigaku Saturn724 CCD diffractometer	1590 independent reflections
Radiation source: rotating anode	1422 reflections with *I* > 2σ(*I*)
multilayer	*R*_int_ = 0.046
Detector resolution: 14.22 pixels mm^-1^	θ_max_ = 27.9°, θ_min_ = 2.7°
ω and φ scans	*h* = −18→19
Absorption correction: multi-scan (*CrystalClear*; Rigaku/MSC, 2005)	*k* = −9→9
*T*_min_ = 0.866, *T*_max_ = 0.914	*l* = −7→7
6462 measured reflections	

### Refinement

**Table:**

Refinement on *F*^2^	Hydrogen site location: inferred from neighbouring sites
Least-squares matrix: full	H-atom parameters constrained
*R*[*F*^2^ > 2σ(*F*^2^)] = 0.026	*w* = 1/[σ^2^(*F*_o_^2^) + (0.0202*P*)^2^] where *P* = (*F*_o_^2^ + 2*F*_c_^2^)/3
*wR*(*F*^2^) = 0.059	(Δ/σ)_max_ = 0.002
*S* = 1.01	Δρ_max_ = 0.33 e Å^−^^3^
1590 reflections	Δρ_min_ = −0.28 e Å^−^^3^
83 parameters	Extinction correction: *SHELXL97* (Sheldrick, 2008), Fc^*^=kFc[1+0.001xFc^2^λ^3^/sin(2θ)]^-1/4^
1 restraint	Extinction coefficient: 0.034 (2)
Primary atom site location: structure-invariant direct methods	Absolute structure: Flack (1983), 716 Friedel pairs
Secondary atom site location: difference Fourier map	Flack parameter: −0.25 (7)

### Special details

**Table:**

Geometry. All e.s.d.'s (except the e.s.d. in the dihedral angle between two l.s. planes) are estimated using the full covariance matrix. The cell e.s.d.'s are taken into account individually in the estimation of e.s.d.'s in distances, angles and torsion angles; correlations between e.s.d.'s in cell parameters are only used when they are defined by crystal symmetry. An approximate (isotropic) treatment of cell e.s.d.'s is used for estimating e.s.d.'s involving l.s. planes.
Refinement. Refinement of *F*^2^ against ALL reflections. The weighted *R*-factor *wR* and goodness of fit *S* are based on *F*^2^, conventional *R*-factors *R* are based on *F*, with *F* set to zero for negative *F*^2^. The threshold expression of *F*^2^ > σ(*F*^2^) is used only for calculating *R*-factors(gt) *etc*. and is not relevant to the choice of reflections for refinement. *R*-factors based on *F*^2^ are statistically about twice as large as those based on *F*, and *R*- factors based on ALL data will be even larger.

### Fractional atomic coordinates and isotropic or equivalent isotropic displacement parameters (Å^2^)

**Table:**

	*x*	*y*	*z*	*U*_iso_*/*U*_eq_	
P1	0.0000	0.74395 (10)	0.33956 (11)	0.01650 (17)	
Cl1	0.0000	0.78689 (13)	0.00139 (10)	0.0391 (3)	
S1	0.0000	0.49265 (10)	0.42063 (18)	0.0294 (2)	
O1	0.07867 (7)	0.86334 (15)	0.4369 (2)	0.0162 (3)	
C1	0.16873 (10)	0.8018 (2)	0.4313 (4)	0.0137 (4)	
C2	0.20060 (11)	0.7047 (2)	0.6162 (3)	0.0166 (4)	
H2	0.1623	0.6729	0.7400	0.020*	
C3	0.29001 (12)	0.6542 (3)	0.6177 (3)	0.0196 (4)	
H3	0.3137	0.5884	0.7442	0.024*	
C4	0.34463 (11)	0.7004 (2)	0.4335 (4)	0.0191 (4)	
H4	0.4056	0.6646	0.4337	0.023*	
C5	0.31099 (12)	0.7976 (3)	0.2507 (3)	0.0203 (5)	
H5	0.3491	0.8291	0.1264	0.024*	
C6	0.22133 (11)	0.8504 (2)	0.2460 (3)	0.0166 (4)	
H6	0.1975	0.9170	0.1204	0.020*	

### Atomic displacement parameters (Å^2^)

**Table:**

	*U*^11^	*U*^22^	*U*^33^	*U*^12^	*U*^13^	*U*^23^
P1	0.0102 (3)	0.0226 (4)	0.0167 (4)	0.000	0.000	−0.0060 (3)
Cl1	0.0210 (4)	0.0820 (7)	0.0144 (4)	0.000	0.000	−0.0040 (4)
S1	0.0152 (3)	0.0179 (3)	0.0549 (5)	0.000	0.000	−0.0040 (4)
O1	0.0084 (6)	0.0174 (7)	0.0228 (7)	−0.0005 (5)	−0.0024 (5)	−0.0040 (6)
C1	0.0078 (8)	0.0143 (9)	0.0188 (9)	−0.0010 (7)	−0.0003 (8)	−0.0051 (8)
C2	0.0150 (9)	0.0197 (12)	0.0151 (11)	−0.0015 (8)	0.0002 (7)	0.0007 (8)
C3	0.0181 (9)	0.0188 (11)	0.0219 (11)	0.0012 (8)	−0.0054 (8)	0.0023 (9)
C4	0.0125 (9)	0.0180 (9)	0.0267 (11)	0.0020 (7)	−0.0026 (8)	−0.0029 (9)
C5	0.0137 (10)	0.0216 (11)	0.0255 (11)	−0.0039 (8)	0.0076 (7)	−0.0015 (8)
C6	0.0162 (10)	0.0163 (10)	0.0174 (10)	−0.0016 (8)	−0.0050 (7)	0.0021 (8)

### Geometric parameters (Å, °)

**Table:**

P1—O1^i^	1.5758 (12)	C2—H2	0.9500
P1—O1	1.5758 (12)	C3—C4	1.390 (3)
P1—S1	1.9114 (11)	C3—H3	0.9500
P1—Cl1	1.9920 (9)	C4—C5	1.378 (3)
O1—C1	1.4234 (17)	C4—H4	0.9500
C1—C2	1.377 (3)	C5—C6	1.398 (2)
C1—C6	1.382 (3)	C5—H5	0.9500
C2—C3	1.390 (2)	C6—H6	0.9500
			
O1^i^—P1—O1	96.79 (9)	C2—C3—C4	119.77 (17)
O1^i^—P1—S1	116.91 (6)	C2—C3—H3	120.1
O1—P1—S1	116.91 (6)	C4—C3—H3	120.1
O1^i^—P1—Cl1	105.42 (6)	C5—C4—C3	120.46 (16)
O1—P1—Cl1	105.42 (6)	C5—C4—H4	119.8
S1—P1—Cl1	113.42 (6)	C3—C4—H4	119.8
C1—O1—P1	121.50 (11)	C4—C5—C6	120.71 (18)
C2—C1—C6	123.07 (15)	C4—C5—H5	119.6
C2—C1—O1	118.43 (16)	C6—C5—H5	119.6
C6—C1—O1	118.41 (17)	C1—C6—C5	117.43 (17)
C1—C2—C3	118.56 (16)	C1—C6—H6	121.3
C1—C2—H2	120.7	C5—C6—H6	121.3
C3—C2—H2	120.7		
			
O1^i^—P1—O1—C1	169.66 (11)	C1—C2—C3—C4	−0.7 (3)
S1—P1—O1—C1	44.80 (16)	C2—C3—C4—C5	0.7 (3)
Cl1—P1—O1—C1	−82.25 (14)	C3—C4—C5—C6	−0.5 (3)
P1—O1—C1—C2	−90.03 (19)	C2—C1—C6—C5	−0.3 (3)
P1—O1—C1—C6	93.23 (17)	O1—C1—C6—C5	176.31 (15)
C6—C1—C2—C3	0.5 (3)	C4—C5—C6—C1	0.2 (3)
O1—C1—C2—C3	−176.05 (16)		

Symmetry codes: (i) −*x*, *y*, *z*.

## References

[bb1] Flack, H. D. (1983). *Acta Cryst.* A**39**, 876–881.

[bb2] Greenhalgh, R., Dhawson, K. L. & Weinberg, P. (1980). *J. Agric. Food Chem.* **28**, 102–105.

[bb3] Rigaku/MSC (2005). *CrystalClear* and *CrystalStructure* Rigaku/MSC Inc. The Woodlands, Texas, USA.

[bb4] Sheldrick, G. M. (2008). *Acta Cryst.* A**64**, 112–122. 10.1107/S0108767307043930 18156677

[bb5] Um, I. H., Jeom, S. E., Baek, M. H. & Dark, H. R. (2003). *Chem. Commun.* **24**, 3016–3017. 10.1039/b310055c 14703835

